# Recent Advances on Familial Hypercholesterolemia in Children and Adolescents

**DOI:** 10.3390/biomedicines10051043

**Published:** 2022-04-30

**Authors:** Francesca Mainieri, Veronica Maria Tagi, Francesco Chiarelli

**Affiliations:** Department of Paediatrics, University of Chieti, 66100 Chieti, Italy; veronicamaria.tagi@alumni.unich.it (V.M.T.); chiarelli@unich.it (F.C.)

**Keywords:** familial hypercholesterolemia, dyslipidemia, low-density lipoprotein cholesterol, atherosclerotic cardiovascular disease, atherosclerosis, treatment, lipid-lowering therapy, children, adolescents

## Abstract

Familial hypercholesterolemia is a common autosomal hereditary disorder characterized by elevated concentrations of low-density lipoprotein cholesterol and the development of premature atherosclerosis and cardiovascular disease. Early diagnosis, as well as prompt and aggressive treatment, are fundamental steps to prevent cardiovascular complications and a high rate of premature mortality in children and adolescents. Clinics and genetics are the two main aspects on which diagnosis is based. Widespread screening programs are a respectable option for the early detection of familial hypercholesterolemia. Different types of screening have been proposed so far; however, the optimal screening program has not yet been found. The treatment approach for both heterozygous and homozygous familial hypercholesterolemia in the pediatric population is multidisciplinary, including lifestyle modifications, standard lipid-lowering medications, and novel pharmacological agents. The latter show promising results, especially for patients who experience intolerance to other treatment or present with more severe conditions. Our purpose is to focus on the importance of the early detection of familial hypercholesterolemia, and to highlight the best therapeutic strategies, including the recent approaches based on current clinical evidence, that need to be adopted from the earliest stages of life.

## 1. Introduction

Familial Hypercholesterolemia (FH) is a genetic condition with high prevalence in the general population around the world; it is characterized by elevated plasma concentrations of cholesterol, in particular high Low-Density Lipoprotein Cholesterol (LDL-C) levels, from birth or even before, possible to detect in utero. People affected by FH show a greater risk of early and progressive atherosclerotic cardiovascular disease (ASCVD), which can also be recognized in children and adolescents, and typical physical signs, such as tendon xanthomas [1]. The hereditary pattern of FH is autosomal dominant; it can be distinguished as a homozygous FH (HoFH), which is caused by a double deleterious mutation in the FH gene and is very rare and severe, and a heterozygous FH (HeFH), which is caused by the mutation of just one allele and is more common, but less severe. In particular, HoFH occurs in one per million individuals worldwide, while two recent meta-analyses have shown that the prevalence of HeFH corresponds to one in around every 311–313 people, with a rough estimate of 6.8–8.5 million children and adolescents affected globally [2,3]. It has been documented that, universally, one baby with HeFH is born per minute [4]. Given the relevance of this disorder, which is considered a current major public health, screening programs and precocious detection of FH have the priority, as reported in 1999 by World Health Organization (WHO) recommendations, and recently confirmed and extended by Wilemon et al. [5]. Nevertheless, diagnosis of FH in the pediatric age is still controversial, since both physical signs and family history might often be difficult to be revealed in youth. Regarding FH treatment in children and adolescents, there is no collective thinking on the best timing, management, and/or types of patients that need it [4]. Therefore, the purpose of this review is to focus on the importance of early FH detection, and to highlight the best therapeutic strategies for both HeFH and HoFH, including the recent approaches based on current clinical evidence, that need to be adopted from the earliest stages of life.

## 2. History and Genetics of Familial Hypercholesterolemia

The main three characteristics that determine FH are elevated blood levels of LDL-C, the presence of tendon xanthomas, and the premature onset of ASCVD. At first, FH was described as a xanthomatous disease, and only by the 1930s was its hereditary nature revealed. The metabolism of LDL was considered the principal target of the several mutations causing the FH phenotype. In particular, Brown and Goldstein had, by the 1970s, identified inherited abnormalities in the LDL receptor (LDLR) in 85–90% of patients, which is considered as the main determinant of this disorder, with results of absent or reduced activity of the accumulation of LDL particles in the plasma [6]. Although rarer, the other genes involved encode for apolipoprotein B (ApoB), protein convertase subtilisin/kexin 9 (PCSK9), and LDLRAP1, the latter being linked to very rare mutations leading to an autosomal recessive form of FH. Specifically, a single mutation of the ApoB gene accounts for approximately 6–10% of all FH cases in the European population. The only common PCSK9 variant in the UK (p.Asp374Tyr) occurs in about 2% of mutation-positive FH patients, while the most common loss-of-function mutation inactivating the PCSK9 protein (p.Arg46Leu) can be found in approximately 3% of European individuals [7]. Alongside the typical HoFH and HeFH forms, there are also compound heterozygous individuals that either show a dissimilar mutation in each allele of the same gene or are double heterozygous, presenting mutations in two different genes that affect LDL-C metabolism [8]. All these different mutations can be implemented into the different residual activity of LDL-R, hence the severity of the disease. Specifically, when the LDL-R gene is mutated, patients might present no LDL-R activity at all, or 2–25% residual activity of LDL-R. Instead, residual activity is seen in the case of ApoB and PCSK9 mutations, and normal LDL-R activity in patients with LDLRAP1 mutations, at the cost of higher blood concentrations of LDL-C, according to a mechanism not yet explained [9].

## 3. Diagnosis

To reach FH diagnosis in children and adolescents, the road that can be followed is based upon two diagnostic principal aspects: clinics and genetics.

### 3.1. Clinical Diagnosis

FH diagnosis relies on numerous clinical diagnostic criteria validated worldwide, including the Dutch Lipid Clinical Network (DLCN) [10], Make Early Diagnosis to Prevent Early Deaths (MEDPED) diagnostic criteria [11], Japan Atherosclerosis Society (JAS) criteria [12], and Simon Broome diagnostic criteria for FH. As stated in the European Society Atherosclerosis (EAS) consensus, the focus of clinical diagnosis is on LDL-C levels and premature (before the age of 55 years in men and 60 years in women) coronary heart disease (CHD) in the family, and/or high LDL-C concentrations and/or genetic mutation for FH in a family member [4]. Generally, according to DLCN, the detection of LDL-C > 330 mg/dL is diagnostic for HoFH. Additionally, when LDL-C blood levels in children are greater than 190 mg/dL on two consecutive visits simultaneously with a 3-month-diet, the diagnosis of FH is more likely. In the case of LDL-C > 160 mg/dL and the concomitance with a family member showing these same LDL-C concentrations (following DLCN criteria in adults and also confirmed by Simon Broome criteria for children younger than 10 years) or premature CHD, FH diagnosis is strongly suggestive [13,14]. If the parent presents a genetic diagnosis of FH, an LDL-C > 130 mg/dL found in the child is indicative of FH [4]. Specifically, an analysis has been recently carried out on a large population sample for the estimate of LDL-C plasma level in children, showing that Sampson’s equation is more reliable than Friedewald’s equation at each considered age class, and even for extreme triglycerides values [15]. However, concerning LDL-C concentrations, each of the diagnostic criteria listed above presents specific ranges in light of the several and possible differences in LDL-C values throughout the pediatric age, especially in adolescence [16,17]. Secondary causes of hypercholesterolemia, such as hypothyroidism, nephrotic syndrome, congenital analbuminemia, weight issues (obesity/anorexia), obstructive liver disease, and hyperlipidemic agents should be promptly excluded [18]. Tendon xanthomata and xanthelasma are clinical features typically found in adults with FH, and more often spotted in children presenting the HoFH rather than HeFH form [19]. The greater part of children and adolescents with HeFH present a completely normal physical examination, with the later appearance of clinical signs [20]. Especially in the case of lack of clinical symptoms, such as in xanthomatosis, it is fundamental to investigate family history for FH and, in this regard, JAS criteria are very useful [12]. Conversely, clinical features in HoFH patients, including tendon and skin xanthomas, deposits of lipids in tendons and joints, usually appear by the first decade of life [21]. Alongside the diagnostic value of xanthomas, they appear as respectable indicators of ASCVD risk. Clinical diagnosis might also be fortified by the presence of arcus corneae, which consists of a white crescentic line due to cholesterol deposition [9].

### 3.2. Genetic Diagnosis

In the pediatric population, the identification of a pathogenic mutation by performing genetic tests, such as Next Generation Sequencing (NGS), is considered the gold standard for the diagnosis of FH [22]. In addition to the HoFH and HeFH forms reported above, individuals with FH can also be categorized into the polygenic form, which is caused by LDL-associated common genetic variations, or the polygenic FH plus hypertriglyceridemia form, which derives from triglyceride-associated mutations together with the LDL-associated ones. Nevertheless, these two last variants are still difficult to detect [23]. As polygenic patients, we can consider those with a clinical diagnosis of FH, but without the identification of a known mutation [24]. Recent findings suggest that, although both monogenic and polygenic FH are associated with an increased CVD risk compared with hypercholesterolemia without an identifiable genetic cause, some differences have been reported. In particular, among individuals with comparable levels of LDL-C, monogenic FH may be linked to a greater risk of CVD, followed by polygenic FH [25]. One possible explanation for this data is that monogenic forms tend to manifest earlier in life, leading to greater cumulative LDL-C exposure [26,27]. It is also possible that polygenic FH responds better than monogenic FH to pharmacological treatments, such as lipid-lowering medication [25]. There is plenty of skepticism about the obligatory execution of genetic tests if the FH phenotype has already been identified, and also because these tests are revealed to be quite expensive. However, genetic results would be a worthy method to identify all the affected people belonging to a specific family, and discover their precise diagnosis [28]. In addition, recent advances in genomics have decreased the costs of genetic testing, improved the understanding of the pathogenesis of FH, and facilitated cascade screening [29]. In some cases, genetic studies might help in selecting a more suitable therapy, that would be then prepared according to the specific phenotype found in patients. An example is the use of the PCSK9 inhibitor, which has been shown to be useful for HeFH patients, but quite ineffective for the HoFH form due to null-type LDL-R mutations [30]. Through genetics, the differential diagnosis between FH and sitosterolemia, a condition that appears with xanthomas, high LDL-C values and a response to dietary intervention can be achieved [31,32,33].

## 4. Screening for FH

### 4.1. Screening for FH

Due to the nature of FH pathophysiology, early diagnosis and treatment are crucial for prevention, especially at a younger age. The identification of increased development of ASCVD in patients with FH prompts the onset of therapeutic approaches and the prevention of premature ASCVD. As stated, ASCVD has been registered in a greater number of patients, as the principal cause of mortality worldwide [34]. Nevertheless, FH sometimes tends to remain underdiagnosed and undertreated. Hence, different types of screening for FH have been proposed so far (Table 1) [35].

Nevertheless, the optimal screening program has not been found due to costs, the privacy of family members of index patients, and organization [36]. Attempts to identify FH when it could be present during the pediatric age are defined as universal screening [37]. Total cholesterol or LDL-C level measured between 1 and 9 years of age best discriminates between individuals with and without FH in the general population [38]. A pilot study confirmed the acceptability of screening for FH with a total cholesterol level at immunization in children aged 1–2 years [39]. Given the hereditary transmission of FH, a cascade screening model has been developed to investigate people belonging to affected patients’ families, in search of LDLR mutations. Currently, at least 1st- and 2nd-degree relatives of the patient should be screened for FH, through lipid panel analysis or genetic testing [40]. In particular, Jackson et al. created a simulation model to simulate multiple family trees starting with progenitor individuals carrying a pathogenic variant for FH who were followed through several generations. Based on this model, cascade genetic testing for FH in the U.S. is cost-effective if started before age 40 in 1st-degree relatives and before age 15 in 2nd-degree relatives [41]. As also demonstrated in several European countries, cascade testing of relatives of those with suspected FH, especially with the monogenic form, is highly cost-effective, since the adoption of cascade services substantially improves quality of life and survival gains [42,43,44,45]. Cascade screening allows the identification of higher numbers of affected patients, and at a premature time, and thus has been strongly suggested around the world. All these precociously detected FH cases are consequently associated with a better prognosis [46]. Frequent scenarios include the detection of normal LDL-C levels and the absence of any mutations in people with physical signs related to a probable clinical diagnosis of FH [47]. In the case of children diagnosed with FH, the subsequent identification of the parents as affected by FH is defined as reverse cascade screening [48]. To achieve the detection targets in FH, a comparison of identification strategies was conducted. According to recent findings, child–parent cascade screening (which integrates cascade screening and child–parent screening) has proved to be the fastest strategy for identifying FH in the population, capable of bridging the FH detection gap [49]. Additional studies on the possibility of adding FH to the heel prick screening of neonates should be conducted, as it would identify affected parents at an early stage [50]. However, ApoB assays, instead of LDL-C and cholesterol levels, would appear more reliable in discriminating affected from healthy neonates [51].

### 4.2. Screening for Subclinical Atherosclerosis and ASCVD

HoFH patients, who are at very high risk of ASCVD, are regularly screened for subclinical atherosclerosis, while this type of screening is not recommended in HeFH individuals because it would not change treatment. Thus, for the first group of people affected, echocardiography of the heart and aorta every year, and coronary CT angiography at least every 5 years, are suggested; the latter is now also available with low-dose radiation for regular use in children [8]. It has also been recently demonstrated that low-dose coronary CT angiography might be used even more frequently due to its higher capability of discovering signs of atherosclerosis, such as the presence of plaque accumulation, than echocardiography [52]. For the assessment of atherosclerosis among both adults and children, and adolescents with FH, carotid intima-media thickness (CIMT) is often considered a surrogate marker, measured by carotid ultrasound scan, which is the most popular, safe, and non-invasive method [53]. Subclinical atherosclerosis at an earlier stage might also be assessed by coronary and/or aortic calcium scores [54]. Particularly, the coronary artery calcium (CAC) score helps in defining risk stratification for ASCVD, typically presenting a heterogeneous clinical course, in HeFH patients. Observation of the presence and possible development of ASCVD in individuals with FH, which usually occurs during adolescence, can be also obtained by the evaluation of arterial stiffness through brachial–ankle pulse wave velocity [55]. Narverud et al. recently studied the immunological and inflammatory pathways involved in early atherosclerosis, showing that LDL-C plays a key role in modulating the expression of several immune-related genes. New data on the involvement of these pathways in early atherosclerosis may represent future therapeutic targets for the prevention of atherosclerotic progression [56]. In the last few decades, several studies have been conducted on the usefulness of novel biochemical cardiovascular biomarkers in risk stratification of children with HF. However, the generalizability of their results is constrained by the small sample sizes and cross-sectional design, not allowing the establishment of prognostic utility. Additionally, the correlation between new biochemical cardiovascular biomarkers and serum cholesterol was not consistently found, so further studies are mandatory [57].

## 5. Management of Familial Hypercholesterolemia

**Figure 1:**
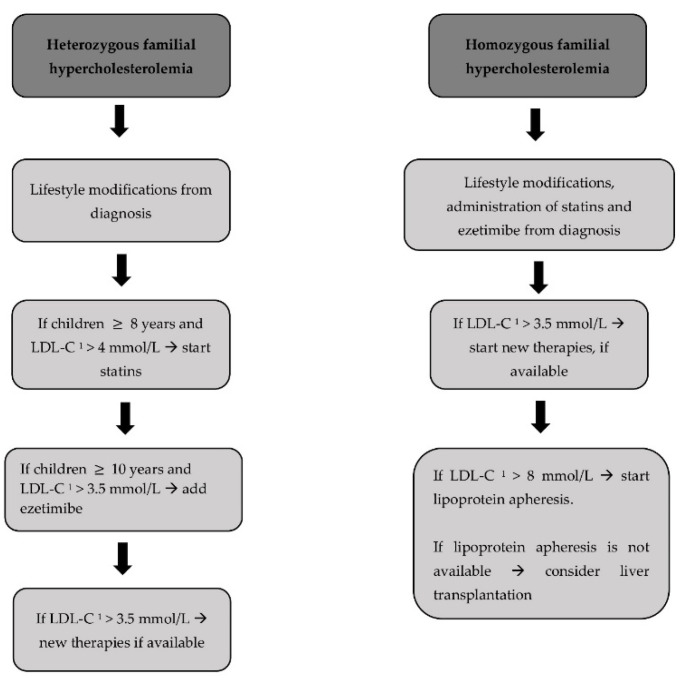
Treatment approach for heterozygous and homozygous familial hypercholesterolemia. LDL-C ^1^, low-density lipoprotein cholesterol.

There are different therapeutic approaches depending on whether HeFH or HoFH needs to be managed (Figure 1).

### 5.1. Treatment for Heterozygous Familial Hypercholesterolemia

#### 5.1.1. Lifestyle Intervention

A healthy diet, namely low in saturated fat and cholesterol, and regular physical activity, are two fundamental steps to gain improvements, even if small, in cholesterol concentrations and preclinical atherosclerotic modifications [58]. In children, the onset of a balanced diet, typically rich in fruits, vegetables, fish, and whole grains, should be after the age of 2 years, thus the involvement of the whole family is further suggested to increase children’s compliance. Regarding calorie distribution, the EAS consensus panel and the National Heart, Lung, Blood Institute (NHLBI) guidelines state that less than 30% calories from total fat, less than 7% calories from saturated fat, and no more than 200 mg of cholesterol should be consumed per day [59]. Cicero et al. recently conducted a pilot clinical study on lipid-lowering dietary programs in a sample of 42 HeFH normal-weight children, demonstrating that dietary intervention to improve food choice, rather than a quantitative diet, seems to be associated with more healthy child behavior [60]. Based on some pediatric studies, the use of plant sterols/stanols (2.2 mg/dL) is considered superior to a low-fat diet alone in significantly lowering LDL-C concentrations. Similarly, guar gum when given as an add-on therapy to bezafibrate reduces total cholesterol and LDL-C levels as compared to bezafibrate as a monotherapy, while poor data have been collected on omega-3 or dietary fiber supplements [61]. Red yeast rice extract and policosanols as dietary supplements have been successfully employed in hypercholesterolemic children, proving to be effective, safe, and well-tolerated in a short-term trial [62]. As previously demonstrated, exercise provides lower lipid levels and risk of cardiovascular diseases by also acting on metabolic conditions, such as obesity, hypertension, and diabetes. Generally, at least 1 h of exercise should be completed by all children, even more by those affected [63]. The earlier the treatment is initiated in HeFH patients, the lower the risk of premature ASCVD. Some lipid-lowering medications have the potential to stop or avoid the occurrence of vascular alterations [64]. These healthy lifestyle regimens should be maintained even after the pharmacological treatment is initiated.

#### 5.1.2. First-Line Lipid-Lowering Therapy

Even though diet is the cornerstone of FH treatment, if considered alone it is not sufficient to maintain desired cholesterol levels. A great improvement in the pharmacological field has been reached through the use of lipid-lowering medications. Nevertheless, FH patients should be constantly monitored, because children who may not need treatment at the time of diagnosis may still have an increased cardiovascular risk and are more likely to require pharmacological treatment in the future. Thus, LDL-C value evaluation should be performed once a year to promptly detect patients with increased LDL-C levels that have to initiate medication. Specifically, children with a genetic diagnosis of FH should start pharmacological treatment when LDL-C levels are > 3.5 mmol/L, while for children with premature cardiovascular disease, or the presence of a family member with high cholesterol levels but without confirmed FH mutation, therapy should be started when LDL-C concentrations are > 4.0 mmol/L [4]. The basis of FH therapy in children and adults is represented by statins. The mechanism of action consists of inhibiting the HMG-CoA reductase enzyme and lowering the biosynthesis of cholesterol in the liver. Furthermore, due to the upregulation of LDL receptors at the cell surface, statins allow the clearance of LDL-C from circulation. In adults, statins confer cardiovascular protection in terms of both primary and secondary prevention and determine a decrease in cardiovascular morbidity and mortality [65]. The European Medicines Agency (EMA) and Food and Drugs Administration (FDA) both approve statins as therapy in children older than 8–10 years of age, including simvastatin, lovastatin, atorvastatin, pravastatin, fluvastatin, and rosuvastatin [66,67]. In particular, the FDA approved pravastatin in children > 8 years of age, while the EMA recognized rosuvastatin as a suitable option for children > 6 years of age. Generally, early statin initiation is recommended, especially for women who will more likely interrupt therapy during pregnancy and lactation [68]. Guidelines suggest starting with a low-dose therapy, with the possibility of an escalation to reach the recommended LDL-C levels [69]. In HeFH children, rosuvastatin led to a reduction of 35–45% in LDL-C levels, a lower worsening of carotid intima-media thickening, and the absence of side effects on growth and sexual development after 2 years of therapy [70]. In patients aged 6–17 years old, pitavastatin showed good results maintaining safety [71]. Due to moderate-to-high-intensity statin treatment, Besseling et al. showed, in a retrospective study, that in patients with HeFH, the risk of coronary artery diseases and mortality was decreased by 44% [72]. More studies are needed to control cardiovascular outcomes obtained with this treatment. In light of the Cochrane review published in 2019 [73], it is clear that statins have the power to decrease mean LDL-C concentrations, at all time points, by a mean of 32% (weighted mean difference of total cholesterol −23% and ApoB −25%) compared to a placebo [74]. Regarding side effects, any important differences in the occurrence of side effects (including rhabdomyolysis or death due to this condition, elevated liver enzymes, creatine kinase, changes in hormones, growth development, electrolyte levels) were observed between children who received the treatment and the placebo group at any time point. As experienced in clinical practice, adults face side effects more often than children, particularly when they are older than 75 years. However, there are no effective observational studies with children, so the prevalence of the adverse effects in the pediatric population after statin therapy has only been estimated, and seems to be lower than in adults [75]. As recently confirmed, statins are safe and generally well-tolerated for adverse effects in the younger population. Furthermore, the evidence that several different medications from the same drug class can be used with similar results supports the pharmacological effect of statins [76]. The efficacy and safety of statins in children and adolescents has been analyzed over short time periods; however, the long-term effects have not been clearly identified so far. Thus, further, long-term, randomized controlled trials of large sample sizes are encouraged to individuate any long-term safety issue that could occur [73]. In the context of efficacy, an aspect not to be underestimated is adherence to therapy, which sometimes can be low due to patients’ lack of awareness of their disease. A confirmed diagnosis, diligent parents acting responsibly regarding their children, and the presence of FH-affected relatives who are used to taking the medications, might help improve treatment adherence [77].

#### 5.1.3. Second-Line Lipid-Lowering Therapy

When statins alone are not able to obtain LDL-C levels within the normal range, different second-line medications can bridge this gap. Ezetimibe represents a widely used lipid-lowering agent with an inhibitory role in the intestinal absorption of dietary and biliary cholesterol; this is achieved by blocking NPC1L1, which decreases the delivery of intestinal cholesterol to the liver [78]. Ezetimibe can be used in children over the age of 10 years, as approved by the EMA and FDA, and it shows good tolerability, efficacy, and long-term safety among young patients in combined administration with statins, as demonstrated in a recent study [79]. Its use has registered a reduction in LDL-C levels below 3.5 mmol/L (<135 mg/dL) in more than 90% of children in double therapy with statins and ezetimibe, compared with only 53% of those on statin therapy only [1]. However, Ezetimibe administered alone is less effective; for example, a randomized controlled trial with ezetimibe monotherapy demonstrated an LDL-C lowering of 27% [80]. Alternative medications might include bile acid sequestrants, niacin, and derivatives of fibric acid; however, the latter two cannot be used in children because of the lack of experience with these medications in this age group, and low tolerability. Regarding bile acid sequestrants, although colesevelam is safe and well-tolerated, its use is only approved by the FDA for children > 10 years of age [81]. These drugs reduce LDL-C by 10–20% by binding bile acids in the intestinal lumen, but may cause substantial gastrointestinal side effects and the reduced absorption of folic acid and fat-soluble vitamins [67].

#### 5.1.4. Third-Line Lipid-Lowering Therapy

In cases where LDL-C goals have not been achieved, the assessment of add-on therapy to appropriate statin treatment, with or without ezetimibe, is necessary. Patients with HeFH can benefit from new monoclonal antibodies targeting PCSK9, specifically evolocumab, currently used in adults and adolescents > 12 years of age with HoFH. However, it is a potential therapy for children with HeFH who have severely elevated LDL-C levels and present intolerance to statins. Evolocumab avoids the degradation of LDL receptors by PCSK9; therefore, LDL-C levels decrease due to the availability of LDL receptors [82]. By performing a randomized, double-blind, placebo-controlled trial, Santos et al. showed that, after 24 weeks of treatment, there was a statistically significant reduction in LDL-C concentrations and secondary lipid variables in the evolocumab group in comparison to the placebo group (−44.5% vs. −6.2%), without important differences in terms of adverse effects [83]. Other medications currently under evaluation include lomitapide, mipomersen, inclisiran, and evinacumab [84]. The evidence of lipid-lowering treatments reducing individual cardiovascular risk is summarized in Table 2.

### 5.2. Treatment for Homozygous Familial Hypercholesterolemia

Given the more severe phenotype of HoFH, an immediate and significant therapeutic approach is mandatory to prevent the incidence of premature ASCVD, most incidences occurring before the age of 30 years.

#### 5.2.1. Lifestyle Modifications

The improvement of daily eating and physical activity habits is essential as the first step for the treatment of HoFH, just as for HeFH patients. A low-saturated fat, low-cholesterol, heart-healthy diet and an active lifestyle are strongly recommended. In case of HoFH, however, even if these adjustments are practiced with strict adherence since the diagnosis, they are not sufficient to treat such a severe form of hypercholesterolemia. Nevertheless, lifestyle modifications have a role in ameliorating the rate of cardiovascular diseases [8].

#### 5.2.2. Lipid-Lowering Agents

A greater delay in clinically evident ASCVD can be achieved by the administration of lipid-lowering therapy. As a matter of fact, the sooner the onset of the treatment, the lower the cardiovascular consequences. However, a broad spectrum of genetic and phenotypic variants of HoFH has been identified, leading to variable responses to standard and new lipid-lowering therapies [8]. Therapy with statins and ezetimibe is usually initiated at diagnosis or within the first year of life, aiming for an LDL-C target lower than 3.5 mmol/L (135 mg/dL), a value that sometimes is not easily achievable with these medications [47]. These targets are ambitious to reach and, additionally, an evaluation of the pharmacological benefits and risks should be carried out before the onset of treatments. The reduction in LDL-C concentrations due to statins and ezetimibe is gained through the upregulation of LDL receptors. Since the most frequent mutation determining HoFH is related to the LDL receptor gene that causes a lack or reduction in LDL receptor levels, the results of such drugs in lowering LDL-C levels are not always satisfactory, as they depend on the residual LDL receptor activity [89]. Hence, even if administered at maximal doses and/or used in conjunction with ezetimibe, bile acid sequestrants, or fibrates, statins are often inadequate to lower LDL-C concentrations in HoFH patients. New additional therapies are therefore required. Some of these medications are approved for the adult population, and are currently under consideration for possible use in children and adolescents. Evolocumab is one of the novel drugs that, similar to statins and ezetimibe, acts on LDL receptors, thus its effect on HeFH patients is greater [30]. In February 2021, the FDA approved evinacumab in adults and children older than 12 years of age, taking into account the results shown by a phase 3 study involving two pediatric patients. Its action consists of angiopoietin-like 3 (ANGPTL3) protein blockage, on the basis that people presenting loss-of-function mutation in ANGPTL3 are hypolipidemic. Furthermore, patients who show two nonsense mutations in LDL receptors may benefit from this novel agent [86]. Another agent that has demonstrated its efficacy on HoFH patients is lomipatide, the target of which is the inhibition of the microsomal triglyceride transport protein (MTTP) in the endoplasmic reticulum of hepatic and intestinal cells, causing a decrease in the secretion of very-low-density lipoprotein (VLDL) cholesterol and chylomicrons into the circulatory system. This is possible because it determines a reduced assembly of lipoproteins that contain ApoB. During the treatment, a balanced low-fat diet is recommended to contrast the severe gastrointestinal side effects and the elevated amount of liver enzymes that may be associated. However, its use has not been approved for children, but has been made available through an extended access program or on a named patients basis, after the approval of the local ethics committee [87]. Another medication approved by the FDA, again only for the adult population, with a warning of hepatotoxicity, is mipomersen, a second-generation antisense oligonucleotide that reduces the translation of ApoB mRNA and the synthesis of ApoB by the ribosome, with the result of decreased VLDL secretion.

#### 5.2.3. Lipoprotein Apheresis

In patients with HoFH, those at high risk of ASCVD and presenting severely elevated LDL-C levels and intolerance to statins, lifestyle modifications and lipid-lowering therapies should be associated with lipoprotein apheresis (LA). Although expensive and time-consuming, it consists of extracorporeal removal of ApoB-containing lipoproteins, such as LDL-C and lipoprotein(a), from circulation. The registered LDL-C reduction after weekly or bi-weekly treatment with LA is around 50–70% in patients showing severe forms of FH [90]. Long-term therapy has proved to result in the regression of xanthomas and atherosclerotic plaques, improving the prognosis of cardiovascular events [9]. Children and adolescents have been treated with LA since 1997, as revealed by the first historical evidence of the use of dextran sulfate cellulose LA on a girl of 4.5 years of age with HoFH and coronary artery disease, with no significant side effects observed. This study suggested the possibility for the early onset of extracorporeal treatment with LA in HoFH children [91]. Based on this evidence, several studies have been conducted that tested LA efficacy and safety in children and adolescents with HoFH, until the FDA approved it for this age group and, finally, it has been considered the most effective treatment at achieving LDL-C goals [92,93,94,95,96]. Thresholds for the onset of the LA procedure vary from country to country [84]. Side effects that might be encountered more frequently are temporary hypotension, abdominal pain, iron and calcium deficiency, fatigue, and port-related sepsis [97].

#### 5.2.4. Rescue Therapy

In severe cases of HoFH where LA is unavailable, and even lipid-lowering treatments are not conclusive in acceptable control of LDL-C levels, liver transplantation needs to be considered. It can achieve a strong and quite direct decrease in LDL-C concentrations (around −80%) [98]. Nevertheless, because of the several related complications and risks, such as high rate of mortality, rarity of compatible donors, possible rejection, and induced immunosuppression, liver transplantation is the last therapeutic resort.

### 5.3. Novel Agents

Various novel drugs can be considered as treatment options for adults with FH, while they have not yet been approved for the affected pediatric population. However, these medications show great potential for their use in the future. Currently, studies are underway for testing the use of alirocumab, a PCSK9 inhibitor, in children and adolescents (8–17 years of age) with both HeFH and HoFH (NCT03510715), with promising results [75,99]. Long-lasting inhibition of PCSK9 synthesis is realized by inclisiran, a small interfering RNA, that can lead to an LDL-C decrease of 47.9%, in comparison with HeFH adults who received a placebo [100]. Its use for the treatment of primary dyslipidemia in adults was approved by the EMA in 2020, while it is still under evaluation for adult patients with HoFH (NCT03851705). As previously mentioned, evolocumab received FDA approval for HoFH patients > 12 years of age, and has also shown its potential as a therapeutic agent for HeFH children with severely increased LDL-C concentrations [83]. In 2020, the FDA approved bempedoic acid, which reduces LDL-C levels through ATP-citrate synthase inhibition, only for the treatment of adult patients with HeFH, on the basis of several studies [101]. While a randomized controlled trial on pediatric patients with HoFH has been undertaken to evaluate the use of mipomersen as adjunctive therapy, with successful results regarding the efficacy of parameters in long-term treatment [88], no similar trials have been conducted on children and adolescents with HeFH.

## 6. Conclusions

FH is a genetic disorder frequently encountered in children and adolescents that leads to high cholesterol levels and markedly elevated LDL-C concentrations. Premature and progressive cardiovascular diseases, clinically evident in childhood and adolescence, have been described as a main consequence of FH. Therefore, as soon as the suspicion of this pathological condition arises, children and adolescents should be referred to specialists. Widespread and well-structured screening programs should be encouraged in order to avoid FH, despite its prevalence and severity, from being underdiagnosed and undertreated. Consequently, both early diagnosis and the early and aggressive initiation of treatment are essential to lower the severely increased LDL-C concentrations and to prevent cardiovascular complications predicted to occur later in life. Alongside the standard lipid-lowering drugs, several novel therapeutic agents with promising results have so far been proposed to achieve better control of LDL-C levels. However, further randomized controlled trials to test new therapeutic strategies for prompt approval are needed, with the ultimate goal of optimizing FH management and prognosis in the pediatric population.

## Figures and Tables

**Table 1:** Different screening methods to detect FH in children and adolescents.

Universal → population screening for a specific age group
Selective → screening for a specific population (usually at high risk)
Cascade → screening from an index case (parent) to family members (including children)
Reverse cascade → screening from an index case (child/adolescent) to other family members
Child–parent → from children screened at a specific age to parents

**Table 2:** Effects of lipid-lowering therapies on individual cardiovascular risk in children and adolescents.

Lifestyle intervention (low-saturated fat and low-cholesterol diet, regular physical activity)	Improvement in cholesterol concentrations. Preclinical atherosclerotic modifications [58]. Ameliorating the rate of cardiovascular diseases [8].
Statins	Significantly less progression of increased CIMT (2-year rosuvastatin treatment) [70]. Children on low-to-moderate intensity statin had a 1% incidence of cardiovascular events after 20-year follow-up. Reduction in LDL-C (32% in HeFH); long-term exposure to lower LDL-C beginning early in life is associated with a substantially greater reduction in the risk of ASCVD. Risk of coronary artery diseases and mortality decreased by 44% [72]. Promotion of CAC progression, possibly reflecting plaque stabilization by reducing the progression of plaque volume and increasing the density of plaque calcium [85].
Ezetimibe	Reduction in LDL-C of 27% in monotherapy; a further 15% reduction in LDL-C when given in double therapy with statins. Improvement in cardiovascular outcomes [80].
Bile acid sequestrants (colesevelam)	Reduction in LDL-C of 10–20% [67].
PCSK9 inhibitors (evolocumab)	Reduction in LDL-C of 38% in HeFH and 21–24% in HoFH, fundamental in preventing underlying ASCVD [30,83].
Evinacumab	Reduction in LDL-C of 49% and cardiovascular events [86].
Lomitapide	Reduction in LDL-C by 58.4% [87]. Decreased secretion of VLDL cholesterol and chylomicrons into the circulatory system.
Mipomersen	Reduction in LDL-C of 42.7% [88]. Decreased VLDL secretion.

## Abbreviations

Familial hypercholesterolemia (FH), low-density lipoprotein cholesterol (LDL-C), atherosclerotic cardiovascular disease (ASCVD), homozygous FH (HoFH), heterozygous FH (HeFH), World Health Organization (WHO), LDL receptor (LDLR), apolipoprotein B (ApoB), Dutch Lipid Clinical Network (DLCN), Make Early Diagnosis to Prevent Early Deaths (MEDPED), Japan Atherosclerosis Society (JAS), European Society Atherosclerosis (EAS), coronary heart disease (CHD), next generation sequencing (NGS), carotid intima-media thickness (CIMT), coronary artery calcium (CAC), National Heart, Lung, Blood Institute (NHLBI), angiopoietin-like 3 (ANGPTL3), microsomal triglyceride transport protein (MTTP), very-low-density lipoprotein (VLDL), lipoprotein apheresis (LA).
